# Flexible Responses to Wildfires by Great Tits ( *Parus major* ) Breeding in Forests of Northeastern Algeria

**DOI:** 10.1002/ece3.73638

**Published:** 2026-05-18

**Authors:** Hocine Mennour, Emilio Pagani‐Núñez, Abdelaziz Bouzegag, Taqiyeddine Bensouilah

**Affiliations:** ^1^ Laboratory of Natural Sciences and Materials, Department of Ecology and Environment, Faculty of Natural and Life Sciences University of Mila Mila Algeria; ^2^ Centre for Conservation and Restoration Science Edinburgh Napier University Edinburgh UK; ^3^ School of Applied Sciences Edinburgh Napier University Edinburgh UK; ^4^ Laboratory of Functional Ecology and Environment University of “Larbi Ben M'hidi” Oum El Bouaghi Oum El Bouaghi Algeria; ^5^ Department of Ecology and Environment, Faculty of Natural and Life Sciences University of Mila Mila Algeria; ^6^ Ecology Department, Faculty of Natural and Life Sciences University of Bordj Bou Arreridj Bordj Bou Arreridj Algeria

**Keywords:** behavioral flexibility, breeding performance, nest composition, *Parus major*, wildfire

## Abstract

Wildfires are increasingly frequent in forests from arid regions due to human activities and climate change, deeply changing vegetation structure and the availability of nesting materials and food resources for breeding birds. There is however a paucity of knowledge about bird responses to wildfires in Mediterranean forests, where the frequency of wildfires is expected to increase due to climate change. To contribute to filling this knowledge gap, we used the Great Tit ( *Parus major* ) as a study model to evaluate nest construction and parental investment in response to post‐fire habitat changes in Mila Province, northeastern Algeria. We conducted the study at three sites in two forests, representing burned (severely impacted by 2019 fires) and unburned (at least since 1997) habitats, in order to assess how these fire regimes shaped reproductive investment across its different stages, from nest construction to fledging success. Nest materials in unburned forests were dominated by moss, while nests in burned areas were constructed with more fibers and contained more dust, reflecting a shift to alternative materials when moss was unavailable. Clutch size correlated positively with the proportion of fibers but was unaffected by fire regime. While eggs were lighter in burned forests, nestling body condition was not directly affected. Hatching success was influenced mainly by first‐egg laying date, with early clutches producing more hatchlings. Notably, fledging success was higher in burned than in unburned forests. Overall, our research shows that wildfires are likely to alter nest materials, but this does not necessarily entail a reduction in the number of fledglings produced. Moreover, despite the high flexibility of Great Tits, their breeding performance was still strongly influenced by seasonal‐related and individual quality factors. By focusing on breeding birds in North Africa, our study provides timely insights into avian resilience to wildfire disturbance in a region where fire risk is projected to increase.

## Graphical abstract

With increasing wildfire frequency in Mediterranean North Africa, this study examines how breeding Great Tits ( *Parus major* ) adjust nest construction and reproductive investment in burned and unburned forests of northeastern Algeria. While fire‐induced scarcity of moss led birds to use alternative fibers and resulted in smaller eggs, fledgling success was paradoxically higher in burned areas, suggesting increased post‐fire food abundance or reduced predation. Our findings demonstrate significant behavioral flexibility in response to habitat alteration, though breeding performance remains ultimately constrained by seasonal and individual quality drivers.

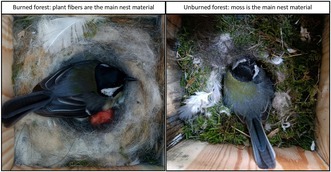

## 1 Introduction

Wildfires are impactful disturbances affecting ecosystems' ecology, altering habitat structure and species distributions (Pausas and Keeley 2009; Kelly et al. 2020). In the Mediterranean basin, there has been an increasing frequency of wildfires driven by climate change and long dry periods (Curt et al. 2020), reducing vegetation cover and habitat suitability for breeding birds (Herrando and Brotons 2002; Brotons et al. 2005). These changes, combined with the immediate impact of wildfires, alter species distributions and lead to declines in bird diversity (Puig‐Gironès et al. 2023; Spatharis et al. 2024). They also affect the breeding ecology and performance of species in several ways, such as altering food availability and predation rates (Wiebe 2014).

Wildfires can alter the breeding ecology of forest birds in various ways, such as by changing the relative abundance of nest construction materials. Nests provide important functions such as insulation, protection from predators, and microclimate regulation (Deeming and Mainwaring 2015). Nest materials and design show variable insulation properties which can influence reproductive success (Windsor et al. 2013; Akresha et al. 2017). Although birds often exhibit species‐specific nest construction behaviors (Alambiaga et al. 2020), they generally use locally available plant material, so that nest composition varies with geographical location (Briggs and Deeming 2016). Thus, changes in forest structure caused by wildfires are likely to affect nest composition materials.

For instance, in urbanized landscapes, bird species rely heavily on anthropogenic materials such as plastic and cigarette butts for nest construction (James Reynolds et al. 2019). In a similar vein, it has been shown that passerine birds such as Blue Tits ( *Cyanistes caeruleus* ) and Great Tits ( *Parus major* ) adjust nest construction by using materials readily available in their proximity, often incorporating unusual materials such as pine needles when the preferred materials are scarce (Lambrechts et al. 2017; Roy et al. 2025). Overall, nest insulation and reproductive success will be maintained within reasonable levels even if birds use alternative nest construction materials (Deeming and Mainwaring 2015).

Our model species, the Great Tit, is a widespread habitat generalist able to persist in highly transformed landscapes (e.g., Ceia et al. 2023), which shows great phenotypic and behavioral flexibility (Charmantier et al. 2008; Husby et al. 2010; Dingemanse et al. 2012). However, given the extensive distribution range of this species, factors associated with nest building may be geographically dependent (Briggs and Deeming 2016), and studies using similarly sized species more closely associated with North African forest ecosystems could potentially yield different ecological inferences. This species relies on specific materials like moss for nest building (Rydgren et al. 2023), with moss content showing a positive relationship with clutch size and hatching success (Álvarez et al. 2013). This dependence on moss could make Great Tits particularly vulnerable to fire‐induced reductions in moss availability (Grover et al. 2020). Additionally, nest size and composition are commonly influenced by temperature and laying date; however, Great Tits appear to be relatively insensitive to these factors (Britt and Deeming 2011). Beyond nest construction, wildfires can influence avian reproduction through their effects on egg traits, offspring development, and overall breeding performance. For instance, Bellia et al. (2011) recorded significant declines in breeding success in recently burned areas, with improvements observed only as vegetation gradually recovered (Bellia et al. 2011).

Furthermore, there is a knowledge gap in animal responses to wildfires which is particularly critical in arid regions of Africa (Geary et al. 2020; Moyo 2022), such as forested landscapes in the southern Mediterranean. Our study aimed thus to fill this gap by studying Great Tit responses to wildfires in northeast Algeria particularly in terms of its impacts on nest construction materials, and how the choice of these materials influenced egg and nesting mass, as well as breeding success. Currently, little is known about how Great Tits respond to wildfires. Since there is a projected increase in the frequency and intensity of wildfires in the Mediterranean area and other arid regions of Africa (Ruffault et al. 2020; Sayedi et al. 2024), it is important to understand how these disturbances affect breeding birds. To this end, we compared the nest construction materials (composition materials and dry mass) and breeding performance (clutch size, hatching success, and fledging success) of Great Tits in burned vs. unburned evergreen forests in northeast Algeria.

We formulated three main predictions. First, we predicted that wildfires would alter nest construction by reducing the availability of preferred materials such as moss, leading Great Tits breeding in burned forests to build nests with lower moss content and increased use of alternative materials, given that moss is the primary nesting material of this species and is known to be sensitive to fire disturbance (Grover et al. 2020). Second, we predicted that these fire‐induced changes in nest design and habitat quality would negatively affect reproductive investment and breeding performance, resulting in lighter eggs and reduced hatching and fledging success in burned compared to unburned forests, as reduced resource availability in post‐fire habitats is expected to constrain maternal investment (Bellia et al. 2011). Finally, we predicted that seasonal constraints would interact with wildfire effects, with later‐breeding pairs showing reduced nest quality and lower reproductive performance, particularly in burned habitats, as seasonal declines in resource availability and individual quality are known to negatively affect breeding performance as the season progresses (Perrins and McCleery 1989; Van Noordwijk et al. 1995). By testing these predictions, this study aims to contribute to our understanding of how birds respond to fire‐induced habitat alterations in Mediterranean forests of North Africa, a region where wildfires are projected to increase in frequency and intensity.

## 2 Materials and Methods

### 2.1 Study Area

**FIGURE 1:**
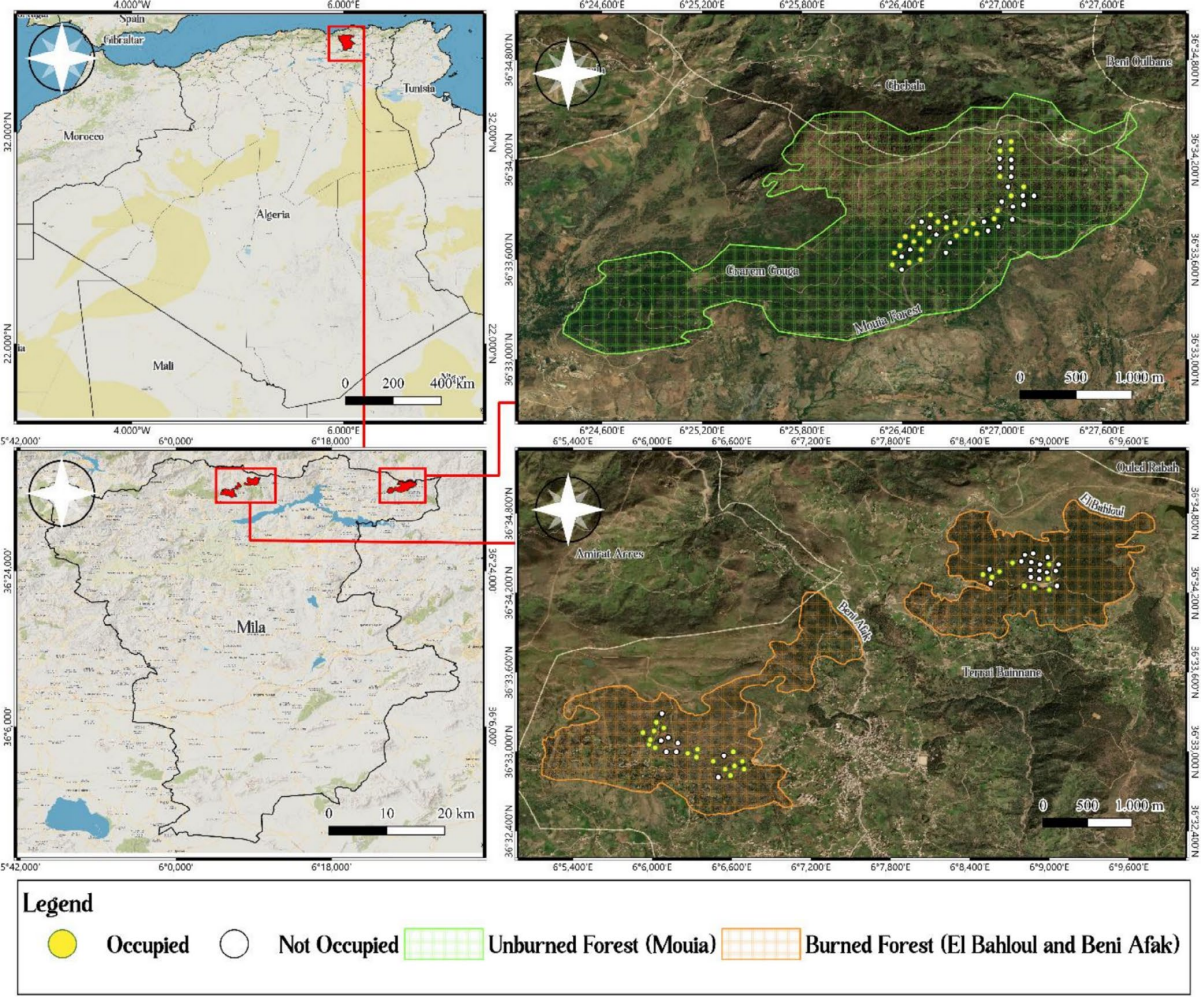
Geographical location of study sites and nestbox distribution in burned and unburned forests of Mila province, Northeastern Algeria.

We conducted this study in three forest stands of the Mediterranean woodlands and forests ecoregion, located in the Tell Atlas mountain range of Mila Province, northeastern Algeria (Figure 1). These stands, share similar Mediterranean climatic conditions (annual precipitation 600–800 mm, hot dry summers 23°C–29°C, cool moist winters 4°C–12°C), comparable elevation ranges (700–1200 m a.s.l.), and were historically dominated by mixed oak forests ( *Quercus suber* and *Q. canariensis*) with similar understory composition. Prior to recent fire events, these forests exhibited comparable structural characteristics including dense canopy cover and well‐developed understory layers.

We selected one fire‐intact reference site and two fire‐affected sites representing contrasting fire regimes. The Mouia Forest (36°33.7’N, 6°26.5′ E; 738 ha) served as our unburned reference. Although this forest experienced wildfires in 1997 and 2009, we selected stands that either escaped recent fire or recovered from the 1997 event, now representing long‐unburnt, late‐seral habitat after nearly 30 years of recovery without fire disturbance (Gana et al. 2024).

The Zouagha Forest (36°31′50.6″N–36°35′1.8″N; 5°59′11″ E–6°10′10″ E; 3915 ha) provided our burned forest treatments. This forest has been severely impacted by recurrent wildfires, with 172 fire incidents recorded between 2011 and 2023 burning 2099.72 ha; the year 2019 was particularly devastating with 564 ha burned (Meghzili et al. 2025; GDF 2024). We selected two stands within this fire‐affected forest, Beni Afak (407 ha; 36°33′N, 6°6′ E; 780–1150 m a.s.l.) and El Bahloul (311.53 ha; 36°34.5′N, 6°9′ E; 729–1102 m a.s.l.), both severely burned in the 2019 wildfires and representing recent, high‐severity fire disturbance with only five years of recovery at the time of our study. The Zouagha forest currently exhibits reduced canopy density and altered understory structure compared to pre‐fire conditions, with vegetation dominated by early‐successional species and resprouting oaks (Meghzili et al. 2025). The unburned site (Mouia Forest) is located approximately 26 km from El Bahloul and 30 km from Beni Afak.

### 2.2 Nest Monitoring

In January 2024, we installed 102 wooden nest boxes across the three study areas, including 52 in unburned forest and 50 in burned forest. The nest boxes used in this study came in two different sizes. The smaller boxes (*n* = 50) had base dimensions of 110 × 110 mm, while the larger ones (*n* = 52) measured 145 × 145 mm. Both designs had an internal height of 230 mm, with a distance of 160 mm from the base to the lower edge of the entrance hole, which had a radius of 32 mm. By including two sizes, we aimed to capture a realistic range of available cavities and to test whether the observed shifts in nest material use (moss vs. fibers) were robust across different cavity sizes, rather than being an artifact of a single box design. This approach is consistent with previous experimental designs in great tit research, notably the study by Demeyrier et al. (2016), who used three box sizes (small, medium, large) to investigate preferences and reproductive consequences in urban environments. Boxes were placed approximately 100 m apart from each other, at a height of 3 m above the ground in order to maximize nesting occupancy rate and ameliorate interspecific competition (Britt and Deeming 2011; Lambrechts et al. 2010, 2013). Nestbox occupation rates and breeding densities were similar between forests (Mouia: 51.92%, 0.090 pairs/ha; Zouagha: 52.00%, 0.087 pairs/ha).

Field surveys were conducted throughout the breeding season (March–June 2024) to monitor reproductive activity, with observations made every 2–3 days. We recorded key breeding parameters, including first egg laying date, clutch size, hatching success (proportion of eggs that hatched, calculated as number of hatchlings divided by clutch size), and fledging success (proportion of eggs that produced fledglings, calculated as number of fledglings divided by clutch size) (Ahola et al. 2009). The laying date of the first egg was determined if a nest contained a single egg upon inspection. This observation date was recorded as the laying date. In cases where multiple eggs were present, the laying date was estimated by subtracting the number of eggs minus one from the observation date, under the assumption that Great Tits lay one egg per day (Mazgajski et al. 2025). The number of hatchlings was determined by counting the number of hatched eggs. We determined fledging success by recording the number of chicks that survived to leave the nest. This typically occurred at 19 to 21 days of age (Bukor et al. 2022; Krams et al. 2025).

### 2.3 Nest Composition

A total of 44 nests of Great Tits were retrieved from the nestboxes at the end of the breeding season across the three forest plots (El Bahloul, *N* = 6; Beni Afak, *N* = 15; and Mouia Forest, *N* = 23 nests). The unequal sample sizes across plots reflected differences in nestbox availability at the end of the season, as some boxes were lost during the breeding season. These nests were placed inside labeled plastic bags and frozen at −20°C for 4 days to eliminate any invertebrates (Briggs and Deeming 2016; Britt and Deeming 2011). After freezing, the nests were dried in a laboratory oven at 60°C for 24 h until constant mass was reached (Glądalski et al. 2024).

To characterize nest composition, we followed the methodology described by Briggs and Deeming (2016). Nests were systematically dismantled to identify and quantify the constituent materials. Each component was weighed with a precision of 0.01 g using a Kern PES 2200‐2 M scale. Unclassified debris was passed through a 0.1 mm mesh sieve to isolate identifiable fragments, while finer particles comprising accumulated nesting and nestling residue were categorized as “dust” and weighed separately. The dry mass included only the structural components of the nest, excluding external elements such as deceased nestlings, to account for material accumulation during the breeding season (Briggs and Deeming 2016; Britt and Deeming 2011). To maintain consistency and minimize observer bias, all measurements were conducted by the same individual (HM).

### 2.4 Egg and Nestling Data Collection

Once full clutches were completed, egg mass was measured using a digital balance to the nearest 0.1 g. Fledgling body condition was assessed once fledglings reached 14–16 days old, with age determined by assessing feathering development (Svensson 1970). Tarsus length, defined as the distance from the inner bend of the tibiotarsal articulation to the base of the toes, was measured using a sliding caliper to the nearest 0.01 mm, and body mass was recorded to the nearest 0.1 g with a digital balance. All measurements were performed by a single person (AB) to ensure consistency and minimize observer bias. Nestling body condition was estimated using the scaled mass index (SMI; Peig and Green 2009). Nestling and egg handling was kept to a minimum, as previous studies have indicated that research activity may negatively impact Great Tit incubation behavior (Clemencin et al. 2024).

### 2.5 Data Analysis

We used linear mixed‐effects models to assess the relationships between nest composition, breeding parameters, and offspring quality in burned and unburned forests.

We first assessed variability in nest material composition across nests. To reduce the dimensionality of nest composition data (14 material types measured in grams), we performed a Principal Component Analysis (PCA) using the *pca* function in the PCAtools package v2.18.0 (Blighe and Lun 2023). Since nest materials were on the same scale, we used raw values in grams to simultaneously capture compositional and size variability (covariance matrix). Prior to analysis, variables with variance below 0.1 were removed. We retained principal components with eigenvalues greater than one in subsequent models. We assessed the effect of forest type (burned or unburned) on these components. To this aim, we ran two generalized linear mixed‐effects models (GLMMs) with the glmmTMB package v1.1.11 (Brooks et al. 2017) using the retained components as response variables, and date (as a continuous variable, number of days since 1st April) and forest type (Unburned vs. Burned) as predictors. We also included the variable nest box size (small or large) but models did not converge well so we finally excluded this variable from these models. We scaled the date variable by subtracting the mean and dividing by standard deviation. Locality (one Mouia site and two Zouagha sites) was the random factor to account for spatial non‐independence of nests within the same area. We ran an additional model with the same structure using scaled total nest mass as a response variable.

We then analyzed the effects of fire regime on egg and nestling condition. We used scaled egg mass and the scaled mass index (SMI) as proxies of egg quality and of nestling condition (Krist 2011; Peig and Green 2009). The SMI is a body condition index that estimates the expected mass of an individual standardized to a reference body size, calculated from the scaling relationship between body mass and a structural size measurement (in our case tarsus length) using standardized major axis regression (Peig and Green 2009). We averaged scores per nest because models using raw values and including nest as a random factor did not converge well. In these two GLMMs, the predictor variables were clutch size (scaled), forest type (burned or unburned), nest box size, first egg laying date (scaled), and nest composition axes (PC1 and PC2), with locality as the random factor. In the models for nestling condition, we used the raw number of hatchlings instead of clutch size.

We finally constructed five separate GLMMs to analyze the effects of nest composition on the different breeding stages. The response variables were clutch size (scaled), number of hatchlings, hatching success (scaled), number of fledglings, and fledging success (scaled). In these three models, predictor variables were first egg laying date (days since April 1st), forest type (burned or unburned), nest box size (small or large), and the two PCA components about nest composition. Locality was included as a random factor.

Spatial autocorrelation in model residuals was assessed using Moran's I (spdep package; Bivand 2022), tested against k‐nearest‐neighbor spatial weights matrices (row‐standardized; *k* = 1–15). To assess robustness, all models were additionally refitted under two spatial structures in glmmTMB (Brooks et al. 2017): an exponential covariance function, with nest coordinates encoded via numFactor, and coordinate‐based random intercepts.

We used a Gaussian error distribution for all models. We verified model assumptions (normality and homoscedasticity of residuals, absence of overdispersion) using DHARMa v0.4.7 and performance v0.15.2 packages (Hartig 2022; Lüdecke et al. 2021). We assessed collinearity among predictors by calculating variance inflation factors (VIFs); all predictor VIFs were below 3, indicating acceptable levels of collinearity (Zuur et al. 2010). We performed all statistical analyses in R v4.4.3 (R Core Team 2023).

## 3 Results

### 3.1 The Effect of Wildfire on Nest Material Composition

**FIGURE 2:**
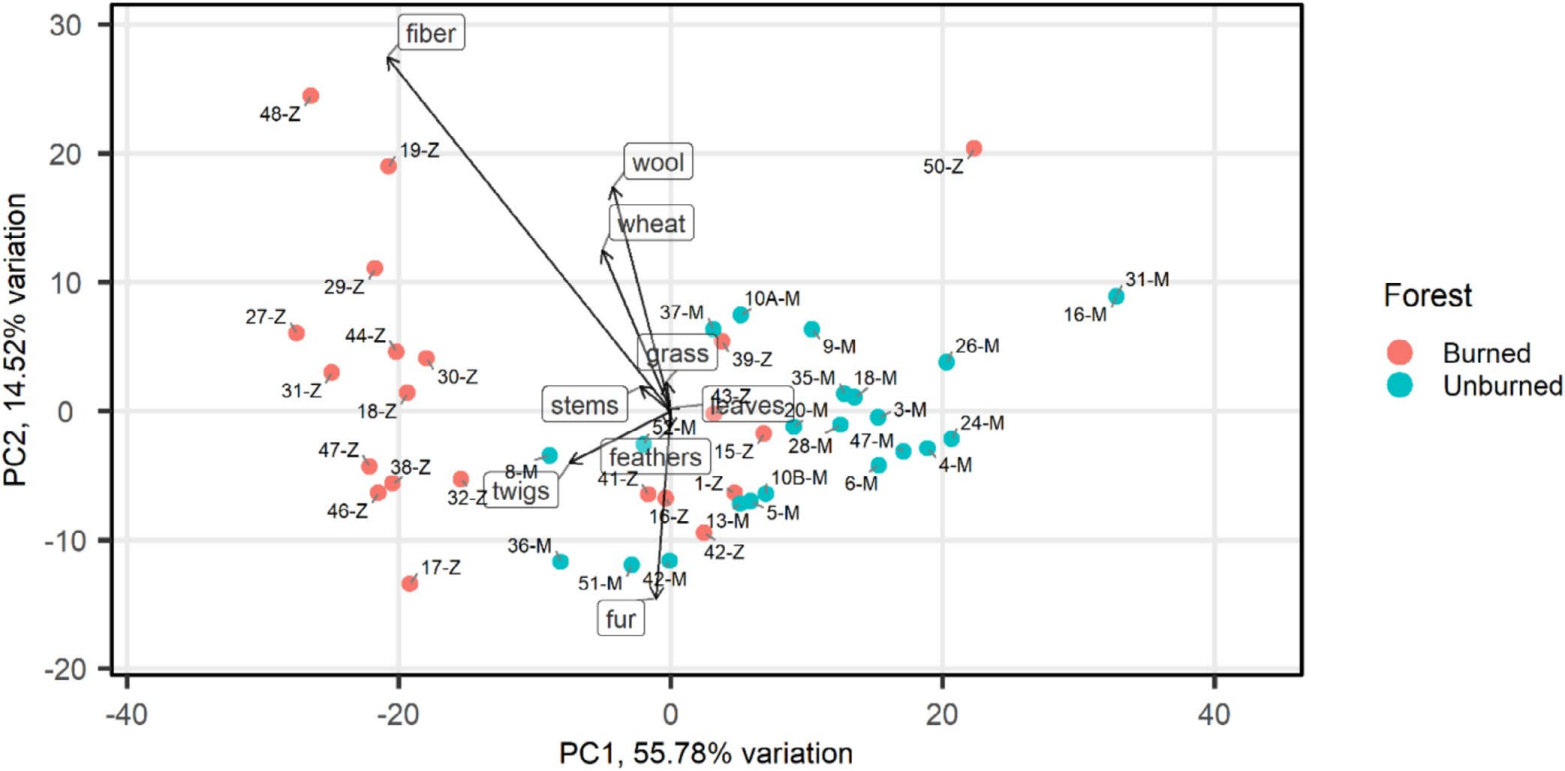
Principal Component Analysis (PCA) biplot of nest material composition. The ordination of nests is displayed along the first two principal components (PC1: 55.79%; PC2: 14.52%). Colored points distinguish forest, with blue indicating the Mouia forest (Unburned) and pink the Zouagha forest (Burned). Vectors depict the loadings of specific nest materials, where direction and length indicate their relative contribution to the variance in nest composition.

The first principal component (PC1) explained 55.79% of the total variance and was dominated by a strong positive loading for moss (loading = 0.91), indicating that moss abundance was the primary driver of variation in nest composition. In contrast, fiber (−0.36) and twigs (−0.13) loaded negatively on PC1, suggesting that these materials were preferentially used when moss was scarce. We thus labeled PC1 as “moss vs fiber”. The second principal component (PC2) accounted for 14.52% of the variance and was characterized by high positive loadings for dust (0.71) and fiber (0.48), and we thus labeled it as “dust and fiber” (Table A1; Figure 2).

For the “moss versus fiber” axis, scores decreased as the breeding season progressed (Table A2). Also, nests in the burned forest had lower scores than those at the unburned forest (Table A2). The “dust and fiber” axis similarly showed a significant negative relationship with laying date, decreasing as the season advanced. Wildfire history had no significant effects on this axis (Table A3).

### 3.2 The Effect of Wildfire on Egg and Nestling Condition

**FIGURE 3:**
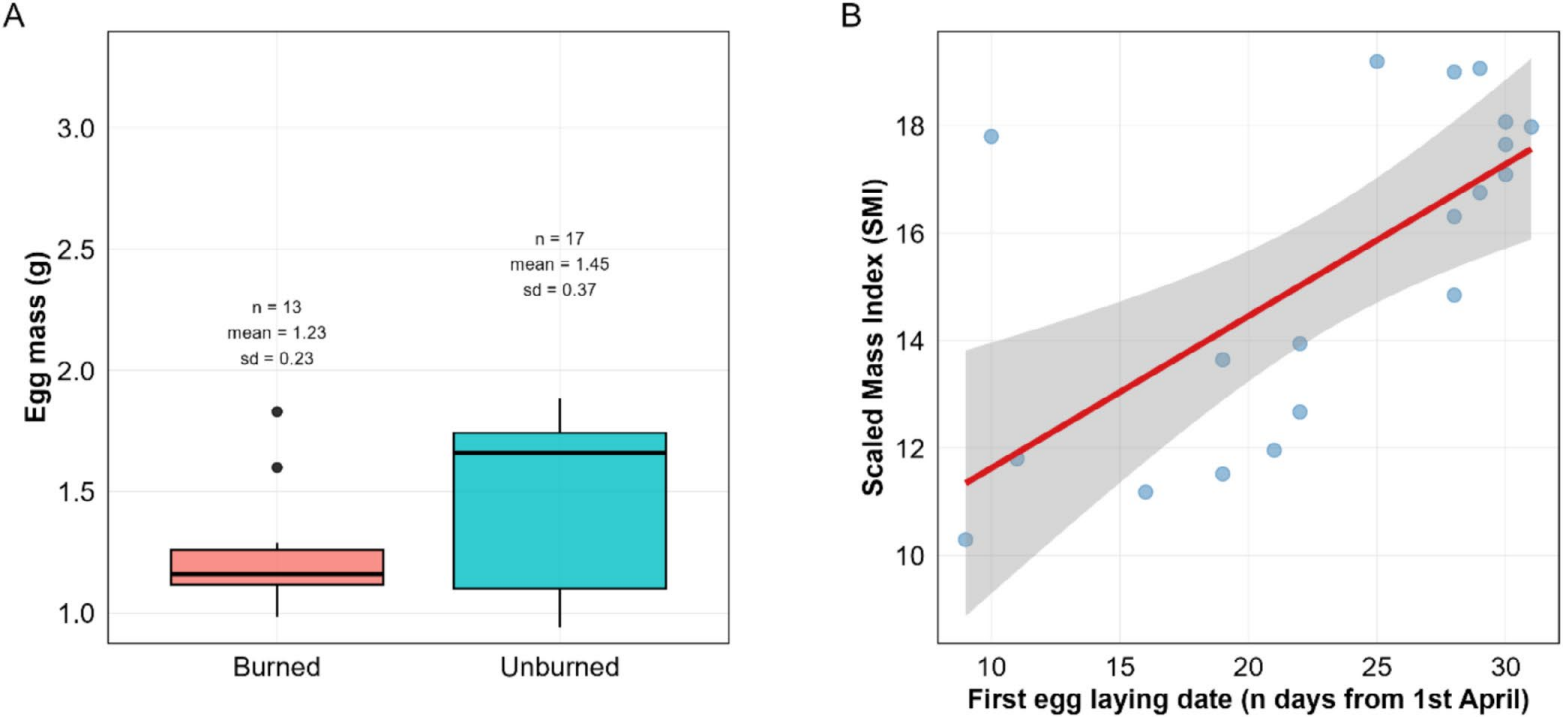
(A) Comparison of egg mass between forest types. The box plot illustrates the variation in egg mass across the two study areas; Mouia (Unburned) and Zouagha (Burned), with eggs being heavier in the unburned forest compared to the burned forest. (B) Seasonal effects on nestling scaled mass index (SMI). The scatter plot illustrates the relationship between laying date (measured as days since April 1st) and nestling body condition (SMI). Blue circles represent individual data points. The solid red line depicts the predicted linear trend, indicating an increase in nestling mass with advancing laying date.

Egg mass was larger in the unburned locality (Mouia) than in the burned ones (two sites at Zouagha) (Burned: *n* = 13, mean = 1.23 g, SD = ±0.23; Unburned: *n* = 17, mean = 1.45 g, SD = ±0.37) (Table A4; Figure 3A). The other variables showed no significant effects.

Nestling scaled mass index (SMI) correlated positively with laying date (Table A5; Figure 3B). The other variables showed no significant effects on nestling condition.

### 3.3 The Effect of Wildfire on Breeding Performance

**FIGURE 4:**
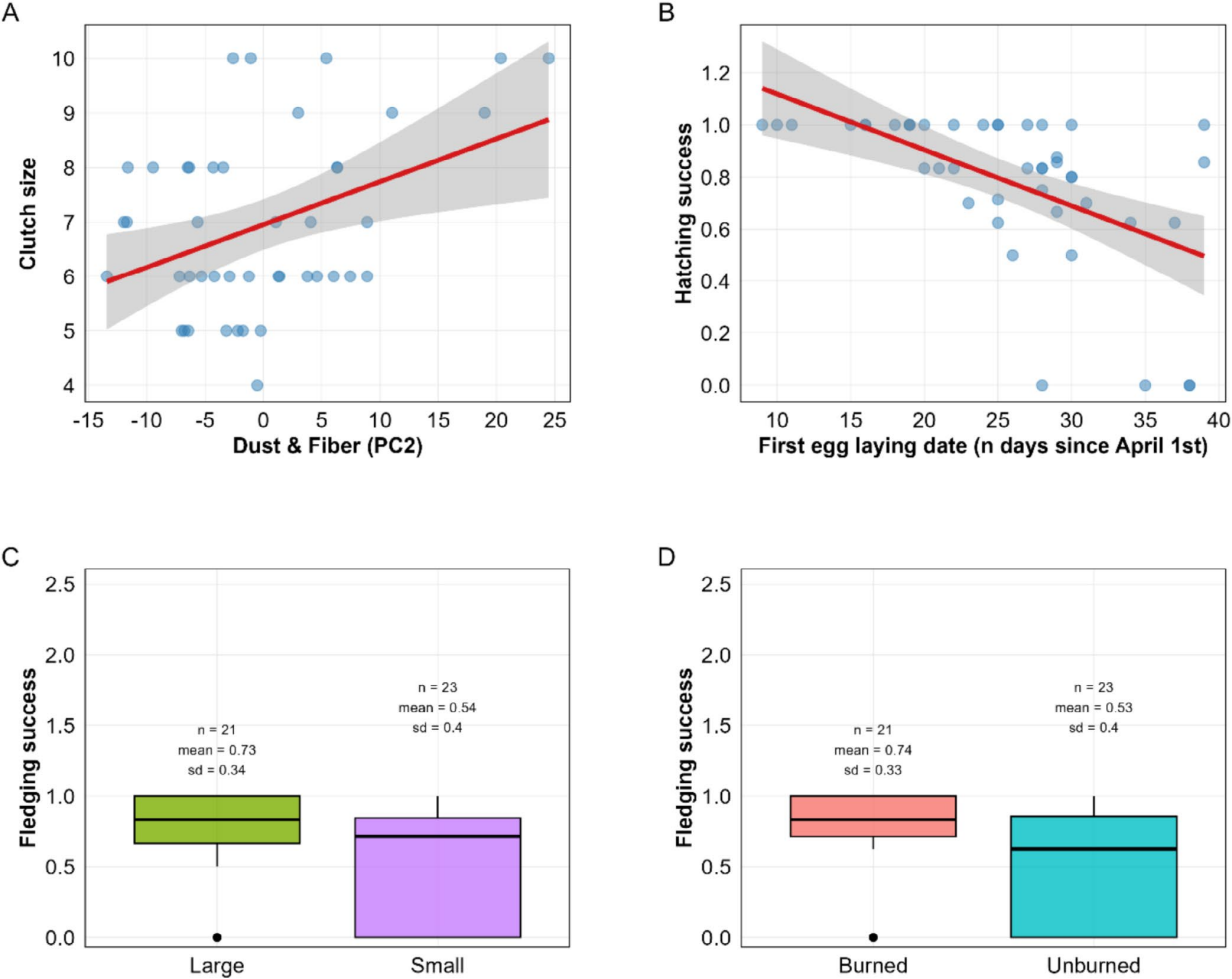
(A) Relationship between clutch size and nest composition. The scatter plot illustrates the positive association between PC2 (Dust/Fiber axis) and clutch size. Blue circles represent individual nest observations. The solid red line depicts the predicted trend from the model. (B) Seasonal decline in hatchling success. The scatter plot illustrates the negative relationship between laying date (measured as the number of days since April 1st) and hatchling success. Blue circles represent individual nest observations. The solid red line depicts the predicted temporal trend, showing a decrease in success as the breeding season progresses. (C) Impact of nest box size on fledging success. The box plot compares fledging success between large and small nestboxes, with large nestboxes having a higher fledging success than small nestboxes. (D) Comparison of fledging success between forests. The box plot displays the variation in fledging success across the two study areas; Mouia (unburned) and Zouagha (burned), with fledging success being higher in the burned forest compared to the unburned forest.

Clutch size only showed a positive correlation with the nest composition variable “dust & fiber” (Table A6; Figure 4A). The other variables showed no significant effects. Hatching success and the number of hatchlings were primarily influenced by laying date, which showed a strong negative effect on both variables (Tables A7 and A8; Figure 4B). No significant effects on clutch size or the number of hatchlings were detected for the other variables. Fledging success and the number of fledglings were significantly higher in the burned than the unburned forest sites (Burned: *n* = 21, mean = 0.74, SD = ±0.33; Unburned: *n* = 23, mean = 0.53, SD = ±0.40) (Burned: mean = 5.52, SD = ±2.80; Unburned: mea*n* = 3.48, SD = ±2.66) and smaller nest boxes were linked to reduced fledging success and fledgling number (Large: *n* = 21, mean = 0.73, SD = ±0.34; Small: *n* = 23, mean = 0.54, SD = ±0.40) (Large: mean = 5.14, SD = ±2.59; Small: mean = 3.83, SD = ±3.05) (Tables A9 and A10; Figure 4C,D). No significant effects were observed for nest composition variables.

### 3.4 Spatial Analysis

Our results showed no spatial autocorrelation in model residuals across any neighborhood size tested. Nine of ten models were non‐significant at all values of *k* (all *p* > 0.09 at *k* = 15; Table A11). The Number of Hatchlings model returned a marginal result at *k* = 2 only (*I* = 0.251, *p* = 0.023), which did not persist at larger neighborhood sizes (*p* = 0.520 at *k* = 15) and is treated as a local artifact. Predictor estimates were consistent in direction, magnitude, and significance across both spatial formulations and the original non‐spatial models (Table A12), with non‐spatial AIC lower or equal in nine of ten cases (Tables A13 and A14). The non‐spatial GLMMs are therefore well justified.

## 4 Discussion

Our results show that Great Tits have high behavioral flexibility in nest construction, selecting alternative materials to moss in burned compared to unburned habitats. They also showed flexibility in parental investment, reducing egg mass but showing higher breeding success in burned compared to unburned habitats. However, the effects of fire regime were not significant in early stages of the breeding season (clutch size, hatching numbers and ratio) and did not influence the second component of nest materials. Thus, Great Tits seemed able to partially compensate for and even benefit from habitat alterations after wildfires. However, parents were unable to fully offset the underlying environmental and seasonal drivers that contributed to shape reproductive investment and success in our study area. Its influence was concentrated at the nest‐building and post‐hatching phases, while early‐stage variables such as clutch size and hatching success remained unaffected regardless of habitat type.

### 4.1 Flexibility in Nest Material Selection in Fire‐Altered Habitats

Consistent with our first prediction, an apparent response to wildfire was a shift in nest material composition. The significant reduction of moss in burned habitats, which was likely driven by its combustion and slow recovery or altered microhabitat conditions (Grover et al. 2020; Fröhlich et al. 2024) seemed to constrain local availability. In unaltered forests, moss is likely the optimal material. First, its high hygroscopicity and structural integrity allow for the construction of a well‐insulated nest cup, providing a stable microclimate essential for early‐season incubation and nestling thermoregulation (Britt and Deeming 2011). Second, moss may confer antiparasitic benefits. In fact, many species contain volatile compounds that could reduce the abundance of nest‐dwelling ectoparasites, a major driver of nestling mortality in Parids (Blunsden and Goodenough 2023). Furthermore, in a stable forest environment, moss is an abundant, spatially aggregated resource, making it highly manageable and efficient to collect. Great Tits compensated by incorporating a higher proportion of natural fibers, suggesting that nest material choice is not a completely fixed species‐specific trait in Great Tits, but a rather flexible behavior shaped by local material availability within a relatively limited radius (Rydgren et al. 2023). This primary moss vs. fiber gradient was structured by both spatial (burned vs. unburned forest) and temporal (seasonal availability) drivers, suggesting that environmental constraints strongly influenced the observed behavior. The second nest composition axis, dominated by fiber and dust, declined with laying date and was positively associated with clutch size, yet it was not influenced by fire regime. Rather than only indicating an active choice, this pattern may also reflect variation in nestling activity and brood size, as dust accumulation is a passive consequence of nestling growth (Britt and Deeming 2011).

### 4.2 Wildfire Alters Reproductive Investment Trade‐Offs Across Breeding Stages

The effects of wildfire on reproduction seemed to be stage‐specific, with our data hinting a trade‐off between initial investment and breeding success. During the egg‐laying stage, females in burned forests produced lighter eggs than in unburned forests. This result suggests reduced maternal investment probably reflecting differences in resource availability during the pre‐laying stage between habitats, and could be linked to population‐level differences in breeding strategies (Riddington and Gosler 1995). This early constraint did not translate into reduced breeding success. Instead, conversely to our second prediction, fledging success was higher in burned forests (see e.g., Mägi et al. 2009). Fire disturbance had no detectable effect on clutch size or hatching success, indicating that early investment decisions remain governed primarily by individual quality and seasonal timing rather than habitat condition. This suggests that post‐fire environments may offer favorable post‐hatching conditions, such as reduced predation or increased insect availability for nestlings, particularly when mediated by microhabitat features like larger nest boxes. Therefore, wildfire appears to reshape the reproductive landscape, constraining initial investment but potentially enhancing offspring survival under certain conditions. While post‐fire environments may occasionally support increased insect availability during the nestling phase (Nappi et al. 2010), our results indicate that such potential benefits do not necessarily translate into enhanced pre‐laying investment.

### 4.3 Persistent Seasonal and Individual Breeding Constraints

We found partial support for our third prediction, that seasonal effects would strongly influence laying date on hatchling numbers and success. However, this pattern seemed consistent across habitats. Phenology and individual quality had pervasive effects that were as important as habitat type in shaping reproductive performance. Early breeding, linked to higher individual quality and better food synchronization (Perrins and McCleery 1989; Van Noordwijk et al. 1995; Culina et al. 2020), was an important determinant of breeding success. This seasonality was also apparent with regards to nest composition. The secondary nest composition component, fiber & dust, seemed to be a consequence of reproductive activity rather than a driver of parental performance. Contrary to the well‐documented seasonal decline in nestling condition (García‐Navas and Sanz 2011), nestling mass appeared to increase with laying date in our study. One possible explanation lies in the temporal coverage of our sample, with only 19 nests and a latest recorded hatching date of 21 May a date approximating the regional caterpillar biomass peak (Ziane et al. 2006). Our data may capture predominantly the pre‐peak portion of the season. García‐Navas and Sanz (2011) have shown that nestling mass tends to increase with hatching date early in the season, a pattern that does not persist after the peak. The unusually late rainfall of early May 2024 may have further delayed both breeding activity and caterpillar emergence (Schöll et al. 2016).

### 4.4 Limitations and Further Research

While our study shows how wildfires affect Great Tit nest construction and reproductive performance, it has apparent limitations. For instance, we did not measure direct drivers of performance such as food availability, parental feeding effort, or nest box microclimatic conditions, which likely contributed to explain differences in egg mass or fledging success (see e.g., Seress et al. 2020). We were also unable to fit some of the models including the variable nest box size. Additionally, nest boxes also have apparent limitations compared to natural nests (Lambrechts et al. 2010). Potential mechanisms driving the observed patterns, such as low predation in burned areas, also remain untested. Nest predation rates were not systematically recorded in our study, and future work should explicitly compare them between burned and unburned forests, given their potential role in explaining the higher fledging success we observed. Future research should test these mechanisms directly. Experiments (e.g., with food supplements or different nest materials) and direct assessment of prey composition, predators, and parental behaviors would shed light on causality. Also, long‐term studies across different stages of habitat recovery are needed to separate immediate fire effects from longer‐term trends. This integrated approach is crucial for predicting how increasing wildfires will impact bird populations.

## 5 Conclusion

In summary, Great Tits respond to fire‐altered habitats displaying high behavioral flexibility during nest‐building and through a trade‐off in reproductive investment across stages. Parents selected fiber materials instead of moss when this apparently was unavailable and produced lighter eggs, which later resulted in higher breeding success in burned compared to unburned forests. Thus, parents successfully compensated for any detrimental effects of wildfires, even reaching relatively high breeding success in burned environments. Yet, this did not prevent them from experiencing strong seasonal effects typical of temperate environments, which ultimately shaped the breeding success of Great Tits in forests of northeast Algeria.

## Author Contributions

**Hocine Mennour:** data curation (equal), formal analysis (equal), investigation (equal), writing – original draft (equal), writing – review and editing (equal). **Emilio Pagani‐Núñez:** conceptualization (equal), data curation (equal), formal analysis (equal), writing – original draft (equal), writing – review and editing (equal). **Abdelaziz Bouzegag:** funding acquisition (equal), investigation (equal), writing – review and editing (equal). **Taqiyeddine Bensouilah:** conceptualization (equal), data curation (equal), formal analysis (equal), funding acquisition (equal), investigation (equal), writing – original draft (equal), writing – review and editing (equal).

## Funding

This work was supported by DGRSDT: la Direction Générale de la Recherche Scientifique et du Développement Technologique (D00L02UN340120230002).

## Ethics Statement

Monitoring, handling and nestbox installation in Mila province, Algeria, were carried out under license 1348/CFM/2024 by the forest conservation agency of Mila province.

## Conflicts of Interest

The authors declare no conflicts of interest.

## Data Availability

Data are available on Dryad at: https://doi.org/10.5061/dryad.t76hdr8g7.

## Appendix A

**TABLE A1:** Principal Component Analysis (PCA) loadings for nest composition.

Variable	PC1	PC2
Nest composition PCA		
Moss	0.91	0.24
Fiber	−0.36	0.48
Dust	−0.03	0.71
Wool	−0.07	0.30
Fur	−0.02	−0.25
Wheat spikes	−0.09	0.22
Twigs	−0.13	−0.07
Feathers	0.00	−0.02
Stems	−0.04	0.03
Grass	−0.01	0.04
Bark	0.00	0.00
Hair	0.00	0.00

*Note:* Values represent loadings for the first two principal components (PC1 and PC2) derived from a covariance matrix (eigenvalues > 1). Nest composition variables represent the dry mass (g) of material types with variance > 0.1; variables falling below this threshold were removed prior to analysis.

**TABLE A2:** Results from a Generalized Linear Mixed Model (GLMM) with a Gaussian distribution examining the factors influencing nest composition (PC1 – Moss‐rich axis) (AIC = 350.7).

Predictor	Variance (Random effect)	Std.Dev. (Random effect)	Β	SE	*z*‐value	*p*‐value	Sig
(Intercept)	41.77	6.46	−10.28	5.32	−1.92	0.05	.
First egg laying Date (scaled)			−4.86	1.99	−2.44	0.01	*
Forest (Unburned vs Burned)			22.40	8.74	2.56	0.01	*

*Note:* The response variable is the first principal component of nest composition (PC1). Predictors included first egg laying date (scaled) and forest status (Unburned vs. Burned). Locality was included as a random factor to account for spatial non‐independence.

**TABLE A3:** Results from a GLMM analyzing the factors influencing nest composition (PC2—Dust and fiber axis) (AIC = 312.6).

Predictor	Variance (Random effect)	Std.Dev. (Random effect)	Β	SE	*z*‐value	*p*‐value	Sig
(Intercept)	< 0.001	0.01	−0.05	1.73	−0.03	0.98	
First egg laying Date (scaled)			−3.60	1.26	−2.85	< 0.01	**
Forest (Unburned vs Burned)			0.10	2.50	0.04	0.97	

*Note:* Predictors included first egg laying date (scaled) and forest status (Unburned vs. Burned), with Locality included as a random factor.

**TABLE A4:** Factors influencing egg mass (g) (AIC = 91.5). Results from a GLMM analyzing the egg mass.

Predictor	Variance (Random effect)	Std.Dev. (Random effect)	Β	SE	*z*‐value	*p*‐value	Sig
(Intercept)	< 0.001	< 0.001	−0.46	0.35	−1.33	0.18	
Clutch size (scaled)			0.30	0.19	1.60	0.11	
Forest (Unburned vs Burned)			1.00	0.43	2.33	0.01	*
Nest Box Size (Small vs large)			−0.16	0.31	−0.51	0.60	
First egg laying Date (scaled)			−0.11	0.28	−0.40	0.68	
Nest Material PC1 (Moss‐rich)			−0.01	0.01	−1.03	0.30	
Nest Material PC2 (Fiber/Dust)			0.01	0.02	0.84	0.39	

*Note:* Predictors included clutch size (scaled), first egg laying date (scaled), forest status (Unburned vs. Burned), nestbox size (Small vs. Large), and nest composition axes (Nest PC1 and PC2). Locality was included as a random factor.

**TABLE A5:** Results from a GLMM analyzing the factors influencing nestling body condition (Scaled Mass Index, SMI; Peig & Green 2009) (AIC = 95.7).

Predictor	Variance (Random effect)	Std.Dev. (Random effect)	Β	SE	*z*‐value	*p*‐value	Sig
(Intercept)	< 0.001	< 0.001	13.87	2.62	5.27	< 0.001	***
Number of Hatchlings	—	—	0.30	0.37	0.82	0.41	
Forest (Unburned vs Burned)	—	—	−0.25	1.59	−0.16	0.87	
Nest Box Size (Small vs large)	—	—	1.19	0.99	1.20	0.22	
First egg laying Date (scaled)	—	—	2.97	0.65	4.50	< 0.001	***
Nest Material PC1 (Moss‐rich)	—	—	−0.02	0.03	−0.80	0.42	
Nest Material PC2 (Fiber/Dust)	—	—	0.05	0.06	0.78	0.43	

*Note:* The dependent variable was the SMI, calculated from nestling body mass and tarsus length measured at day 14–16, using a Standardized Major Axis (SMA) regression slope. Predictors included brood size, first egg laying date (April‐based, scaled), forest status (burned vs. unburned), nestbox size (small vs. large), and nest composition axes (PC1: moss‐rich axis; PC2: dust & fiber axis). Locality was included as a random factor.

**TABLE A6:** Results from a GLMM analyzing the factors influencing clutch size (AIC = 126.7).

Predictor	Variance (Random effect)	Std.Dev. (Random effect)	Β	SE	*z*‐value	*p*‐value	Sig
(Intercept)	< 0.001	< 0.001	0.20	0.29	0.77	0.43	
First egg laying Date (scaled)	—	—	0.17	0.15	1.10	0.27	
Forest (Unburned vs Burned)	—	—	−0.20	0.39	−0.53	0.60	
Nest Box Size (Small vs large)	—	—	−0.23	0.27	−0.85	0.39	
Nest Material PC1 (Moss‐rich)	—	—	−0.01	0.01	−1.10	0.27	
Nest Material PC2 (Fiber/Dust)	—	—	0.05	0.02	3.19	< 0.01	**

*Note:* Predictors included first egg laying date (scaled), forest status (Unburned vs. Burned), nestbox size (Small vs. Large), and nest composition axes (Nest PC1 and PC2). Locality was included as a random factor.

**TABLE A7:** Results from a GLMM analyzing the factors influencing the number of hatchlings per nest (AIC = 200.8).

Predictor	Variance (Random effect)	Std.Dev. (Random effect)	Β	SE	*z*‐value	*p*‐value	Sig
(Intercept)	< 0.01	0.04	5.68	0.67	8.43	< 0.001	***
First egg laying Date (scaled)	—	—	−1.05	0.37	−2.83	< 0.01	**
Forest (Unburned vs Burned)	—	—	0.35	0.90	0.39	0.70	
Nest Box Size (Small vs large)	—	—	−0.67	0.61	−1.09	0.28	
Nest Material PC1 (Moss‐rich)	—	—	−0.04	0.02	−1.55	0.12	
Nest Material PC2 (Fiber/Dust)	—	—	0.05	0.04	1.35	0.18	

*Note:* Predictors included first egg laying date (scaled), forest status (Unburned vs. Burned), nestbox size (Small vs. Large), and nest composition axes (Nest PC1 and PC2). Locality was included as a random factor.

**TABLE A8:** Results from a GLMM analyzing the factors influencing hatching success (number of hatchlings divided by clutch size) (AIC = 119.6).

Predictor	Variance (Random effect)	Std.Dev. (Random effect)	Β	SE	*z*‐value	*p*‐value	Sig
(Intercept)	< 0.001	< 0.01	0.05	0.26	0.19	0.85	
First egg laying Date (scaled)	—	—	−0.68	0.15	−4.63	< 0.001	***
Forest (Unburned vs Burned)	—	—	0.40	0.36	1.11	0.27	
Nest Box Size (Small vs large)	—	—	−0.30	0.24	−1.22	0.22	
Nest Material PC1 (Moss‐rich)	—	—	−0.01	0.01	−1.30	0.20	
Nest Material PC2 (Fiber/Dust)	—	—	−0.01	0.02	−0.62	0.54	

*Note:* Predictors included first egg laying date (scaled), forest status (Unburned vs. Burned), nestbox size (Small vs. Large), and nest composition axes (Nest PC1 and PC2). Locality was included as a random factor.

**TABLE A9:** Results from a GLMM analyzing the factors influencing the number of fledglings, i.e., the total number of nestlings that successfully fledged (AIC = 219.3).

Predictor	Variance (Random effect)	Std.Dev. (Random effect)	Β	SE	*z*‐value	*p*‐value	Sig
(Intercept)	< 0.001	< 0.01	6.86	0.83	8.24	< 0.001	***
First egg laying Date (scaled)	—	—	−0.09	0.46	−0.20	0.85	
Forest (Unburned vs Burned)	—	—	−2.77	1.11	−2.50	0.01	*
Nest Box Size (Small vs large)	—	—	−1.83	0.76	−2.41	0.02	*
Nest Material PC1 (Moss‐rich)	—	—	0.03	0.03	0.94	0.35	
Nest Material PC2 (Fiber/Dust)	—	—	0.05	0.05	1.11	0.27	

*Note:* Predictors included First egg laying date (scaled), forest status (Unburned vs. Burned), nestbox size (Small vs. Large), and nest composition axes (Nest PC1 and PC2). Locality was included as a random factor.

**TABLE A10:** Factors influencing fledging success (AIC = 128.3).

Predictor	Variance (Random effect)	Std.Dev. (Random effect)	Β	SE	*z*‐value	*p*‐value	Sig
(Intercept)	< 0.001	< 0.001	0.85	0.29	2.86	0.004	**
First egg laying Date (scaled)	—	—	−0.11	0.16	−0.68	0.50	
Forest (Unburned vs Burned)	—	—	−0.93	0.39	−2.35	0.02	*
Nest Box Size (Small vs large)	—	—	−0.69	0.27	−2.57	0.01	*
Nest Material PC1 (Moss‐rich)	—	—	0.02	0.01	1.39	0.16	
Nest Material PC2 (Fiber/Dust)	—	—	< 0.001	0.02	0.002	0.99	

*Note:* Results from a GLMM analyzing fledging success (number of fledglings divided by clutch size). Predictors included first egg laying date (scaled), forest status (Unburned vs. Burned), nestbox size (Small vs. Large), and nest composition axes (Nest PC1 and PC2). Locality was included as a random factor.

**TABLE A11:** Moran's I sensitivity results (k = 1–15).

Model	*n*	First sig. k	I (sig. k)	*p* (sig. k)	Last k	I (last k)	*p* (last k)	Result
I—Nest Composition								
Nest Material PC1 (Moss‐rich)	44	—	—	—	15	−0.058	0.804	OK
Nest Material PC2 (Fiber/Dust)	44	—	—	—	15	−0.054	0.781	OK
Nest Mass	44	—	—	—	15	−0.060	0.823	OK
II—Breeding Performance								
Clutch Size	44	—	—	—	15	−0.039	0.650	OK
Number of Hatchlings	44	2	+0.251	0.023	15	−0.025	0.520	k = 2 artefact
Hatching Success	44	—	—	—	15	−0.036	0.629	OK
Number of Fledglings	44	—	—	—	15	−0.069	0.874	OK
Fledging success	44	—	—	—	15	−0.069	0.873	OK
III—Egg & Nestling Condition								
Egg Mass	30	—	—	—	15	−0.085	0.879	OK
Scaled Mass Index (SMI)	19	—	—	—	15	−0.067	0.711	OK

*Note:* First sig. k = first neighborhood size at which *p* < 0.05; — = never significant.

**TABLE A12:** Model estimates comparison across spatial models.

Predictor	Non‐Spatial *β* [SE], *z*, *p*	exp(pos) *β* [SE], *z*, *p*	x/y *β* [SE], *z*, *p*
I. Nest Composition—Nest Material PC1 (Moss‐rich)			
First egg laying Date (scaled)	−4.864 [1.999], −2.43, 0.015*	−4.689 [2.014], −2.33, 0.020*	−3.651 [1.892], −1.93, 0.054 .
Forest (Unburned vs Burned)	+22.406 [8.743], 2.56, 0.010*	+23.215 [7.851], 2.96, 0.003**	+22.323 [4.558], 4.90, < 0.001***
I. Nest Composition—Nest Material PC2 (Fiber/Dust)			
First egg laying Date (scaled)	−3.595 [1.261], −2.85, 0.004**	−3.593 [1.261], −2.85, 0.004**	−3.593 [1.261], −2.85, 0.004**
Forest (Unburned vs Burned)	−0.103 [2.495], −0.04, 0.967	−0.112 [2.495], −0.05, 0.964	−0.112 [2.495], −0.05, 0.964
I. Nest Composition—Nest Mass			
First egg laying Date (scaled)	−0.181 [0.161], −1.13, 0.259	−0.181 [0.161], −1.13, 0.259	−0.179 [0.161], −1.12, 0.264
Forest (Unburned vs Burned)	−0.188 [0.318], −0.59, 0.554	−0.188 [0.318], −0.59, 0.554	−0.170 [0.343], −0.50, 0.620
II. Breeding Performance—Clutch Size			
First egg laying Date (scaled)	+0.175 [0.159], 1.10, 0.270	+0.175 [0.159], 1.10, 0.270	+0.176 [0.185], 0.95, 0.341
Forest (Unburned vs Burned)	−0.205 [0.387], −0.53, 0.596	−0.205 [0.387], −0.53, 0.596	−0.012 [0.479], −0.03, 0.979
Nest Box Size (Small vs large)	−0.225 [0.265], −0.85, 0.395	−0.225 [0.265], −0.85, 0.395	−0.209 [0.263], −0.79, 0.428
Nest Material PC1 (Moss−rich)	−0.012 [0.011], −1.10, 0.272	−0.012 [0.011], −1.10, 0.272	−0.019 [0.013], −1.50, 0.134
Nest Material PC2 (Dust/Fiber)	+0.055 [0.017], 3.19, 0.001**	+0.055 [0.017], 3.19, 0.001**	+0.062 [0.018], 3.46, < 0.001***
II. Breeding Performance—Number of Hatchlings			
First egg laying Date (scaled)	−1.042 [0.369], −2.83, 0.005**	−1.042 [0.369], −2.83, 0.005**	−0.760 [0.437], −1.74, 0.082 .
Forest (Unburned vs Burned)	+0.357 [0.896], 0.40, 0.691	+0.357 [0.896], 0.40, 0.691	+0.628 [1.053], 0.60, 0.551
Nest Box Size (Small vs large)	−0.666 [0.615], −1.08, 0.278	−0.666 [0.615], −1.08, 0.278	−0.263 [0.705], −0.37, 0.709
Nest Material PC1 (Moss‐rich)	−0.039 [0.025], −1.56, 0.118	−0.039 [0.025], −1.56, 0.118	−0.054 [0.029], −1.88, 0.060 .
Nest Material PC2 (Dust/Fiber)	+0.054 [0.040], 1.35, 0.176	+0.054 [0.040], 1.35, 0.176	+0.078 [0.044], 1.80, 0.072 .
II. Breeding Performance—Hatching Success			
First egg laying Date (scaled)	−0.679 [0.147], −4.63, < 0.001***	−0.679 [0.147], −4.63, < 0.001***	−0.572 [0.174], −3.28, 0.001**
Forest (Unburned vs Burned)	+0.396 [0.357], 1.11, 0.267	+0.396 [0.356], 1.11, 0.267	+0.433 [0.402], 1.08, 0.281
Nest Box Size (Small vs large)	−0.299 [0.244], −1.22, 0.221	−0.299 [0.244], −1.22, 0.221	−0.111 [0.302], −0.37, 0.715
Nest Material PC1 (Moss−rich)	−0.013 [0.010], −1.30, 0.195	−0.013 [0.010], −1.29, 0.195	−0.016 [0.011], −1.50, 0.133
Nest Material PC2 (Dust/Fiber)	−0.010 [0.016], −0.62, 0.538	−0.010 [0.016], −0.62, 0.538	−0.004 [0.016], −0.25, 0.803
II. Breeding Performance—Number of Fledglings			
First egg laying Date (scaled)	−0.088 [0.455], −0.19, 0.847	−0.088 [0.455], −0.19, 0.846	−0.255 [0.535], −0.48, 0.634
Forest (Unburned vs Burned)	−2.772 [1.107], −2.51, 0.012*	−2.770 [1.107], −2.50, 0.012*	−2.754 [1.129], −2.44, 0.015*
Nest Box Size (Small vs large)	−1.830 [0.759], −2.41, 0.016*	−1.829 [0.759], −2.41, 0.016*	−1.997 [0.804], −2.48, 0.013*
Nest Material PC1 (Moss−rich)	+0.029 [0.031], 0.94, 0.346	+0.029 [0.031], 0.94, 0.346	+0.031 [0.031], 1.00, 0.316
Nest Material PC2 (Dust/Fiber)	+0.055 [0.049], 1.11, 0.266	+0.055 [0.049], 1.11, 0.266	+0.050 [0.049], 1.03, 0.302
II. Breeding Performance—Fledging success			
First egg laying Date (scaled)	−0.109 [0.162], −0.68, 0.499	−0.109 [0.162], −0.68, 0.499	−0.095 [0.227], −0.42, 0.674
Forest (Unburned vs Burned)	−0.925 [0.393], −2.35, 0.019*	−0.925 [0.393], −2.35, 0.019*	−0.924 [0.400], −2.31, 0.021*
Nest Box Size (Small vs large)	−0.693 [0.270], −2.57, 0.010*	−0.693 [0.270], −2.57, 0.010*	−0.673 [0.352], −1.91, 0.056 .
Nest Material PC1 (Moss‐rich)	+0.015 [0.011], 1.40, 0.163	+0.015 [0.011], 1.40, 0.163	+0.015 [0.011], 1.39, 0.165
Nest Material PC2 (Dust/Fiber)	+0.000 [0.017], 0.002, 0.998	+0.000 [0.017], 0.002, 0.998	+0.000 [0.018], 0.017, 0.987
III. Egg & Nestling Condition—Egg Mass			
Clutch Size (scaled)	+0.308 [0.193], 1.60, 0.110	+0.308 [0.193], 1.60, 0.110	+0.267 [0.171], 1.56, 0.118
Forest (Unburned vs Burned)	+1.010 [0.433], 2.33, 0.020*	+1.010 [0.433], 2.33, 0.020*	+1.389 [0.501], 2.77, 0.006**
Nest Box Size (Small vs large)	−0.166 [0.319], −0.52, 0.604	−0.166 [0.319], −0.52, 0.604	−0.171 [0.241], −0.71, 0.477
First egg laying Date (scaled)	−0.115 [0.281], −0.41, 0.683	−0.115 [0.281], −0.41, 0.683	+0.061 [0.203], 0.30, 0.764
Nest Material PC1 (Moss‐rich)	−0.014 [0.014], −1.04, 0.301	−0.014 [0.014], −1.04, 0.301	−0.029 [0.013], −2.27, 0.023*
Nest Material PC2 (Dust/Fiber)	+0.019 [0.023], 0.85, 0.396	+0.019 [0.023], 0.85, 0.396	+0.027 [0.020], 1.36, 0.174
III. Egg & Nestling Condition—Scaled Mass Index (SMI)			
Number of Hatchlings	+0.310 [0.377], 0.82, 0.412	No convergence	No convergence
Forest (Unburned vs Burned)	−0.255 [1.590], −0.16, 0.872	No convergence	No convergence
Nest Box Size (Small vs large)	+1.200 [0.994], 1.21, 0.227	No convergence	No convergence
First egg laying Date (scaled)	+2.970 [0.659], 4.51, < 0.001***	No convergence	No convergence
Nest Material PC1 (Moss‐rich)	−0.029 [0.036], −0.80, 0.423	No convergence	No convergence
Nest Material PC2 (Dust/Fiber)	+0.052 [0.066], 0.78, 0.434	No convergence	No convergence

*Note:* No convergence = model failed to produce valid estimates under that spatial structure. Significance codes: *** *p* < 0.001, ** *p* < 0.01, * *p* < 0.05, . *p* < 0.1.

**TABLE A13:** AIC comparison across spatial formulations.

Model	Non‐Spatial AIC	exp(pos) AIC	*x*/*y* AIC
I. Nest Composition			
Nest Material PC1 (Moss‐rich)	350.7	353.4	NA (no convergence)
Nest Material PC2 (Fiber/Dust)	312.6	314.6	314.6
Nest Mass	131.3	NA (no convergence)	132.9
II. Breeding Performance			
Clutch Size	126.7	NA (no convergence)	127.4
Number of Hatchlings	200.8	NA (no convergence)	201.3
Hatching Success	119.6	121.6	120.4
Number of Fledglings	219.3	NA (no convergence)	221.0
Fledging success	128.3	NA (no convergence)	130.3
III. Egg & Nestling Condition			
Egg Mass	91.5	93.5	85.3
Scaled Mass Index (SMI)	95.7	NA (no convergence)	NA (no convergence)

*Note:* NA (no convergence) = model failed to converge under that spatial structure.

**TABLE A14:** Model convergence diagnostic comparison.

Model	Non‐Spatial Conv.	exp(pos) Conv.	*x*/*y* Conv.
I. Nest Composition			
Nest Material PC1 (Moss‐rich)	OK	OK	Failed
Nest Material PC2 (Fiber/Dust)	OK	OK	OK
Nest Mass	OK	Failed	OK
II. Breeding Performance			
Clutch Size	OK	Failed	OK
Number of Hatchlings	OK	Failed	OK
Hatching Success	OK	OK	OK
Number of Fledglings	OK	Failed	OK
Fledging success	OK	Failed	OK
III. Egg & Nestling Condition			
Egg Mass	OK	OK	OK
Scaled Mass Index (SMI)	OK	Failed	Failed

*Note:* OK = converged successfully; Failed = non‐positive‐definite Hessian matrix or false convergence.
